# Supplementary material for: Quinuclidinium salt ferroelectric thin-film with duodecuple-rotational polarization-directions
Source: Nat Commun. 2017 Apr 4;8:14934. doi: 10.1038/ncomms14934 (PMC5382275; doi:10.1038/ncomms14934)
Supplement: Supplementary Information — Supplementary Figures, Supplementary Tables, Supplementary Note, Supplementary Method and Supplementary References [file ncomms14934-s1.pdf]

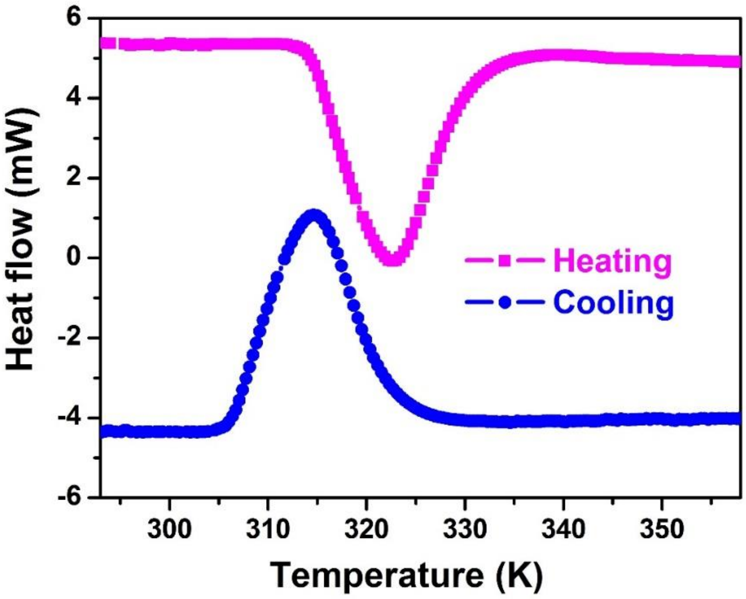

Supplementary Figure 1. DSC curves for 1. The thermal anomalies reveal a reversible phase transition.

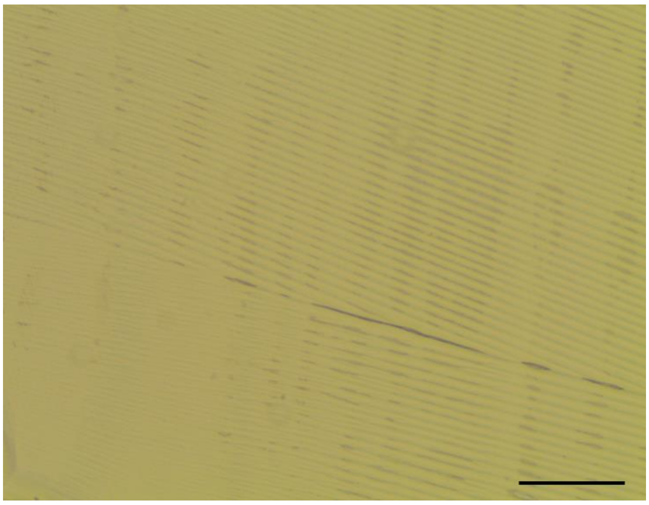

Supplementary Figure 2. Optical microscope photograph of the thin-film of 1. The film was deposited on the ITO-coated glass. (Scale bar: 100 μm)

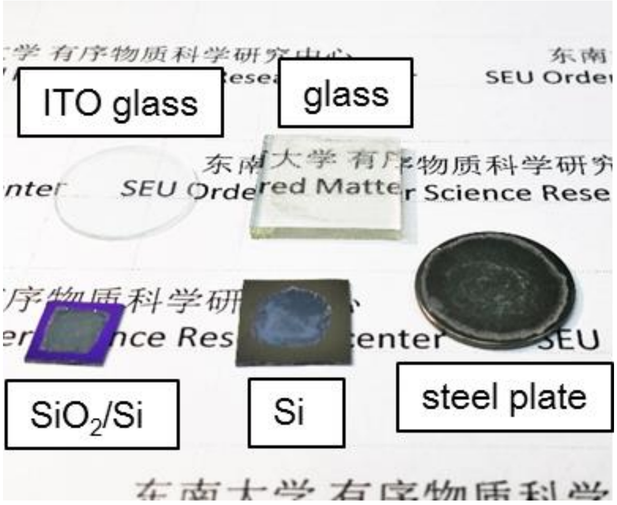

Supplementary Figure 3. Photo of thin-films of 1 on different substrates.

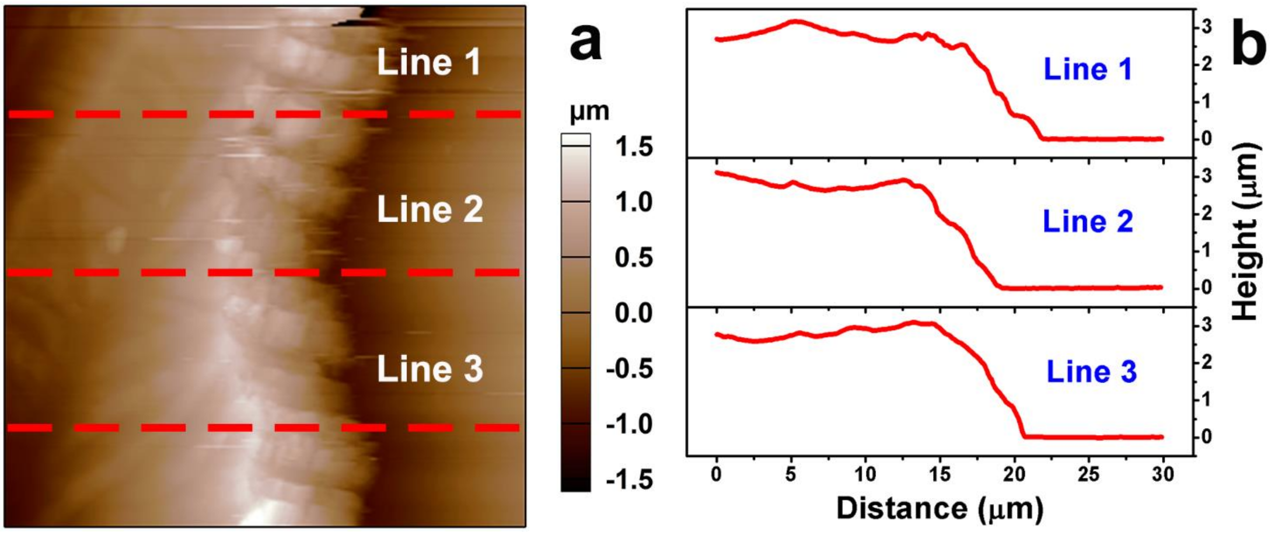

Supplementary Figure 4. AFM image of the crystal of 1. (a) AFM images. (b) Height profiles.

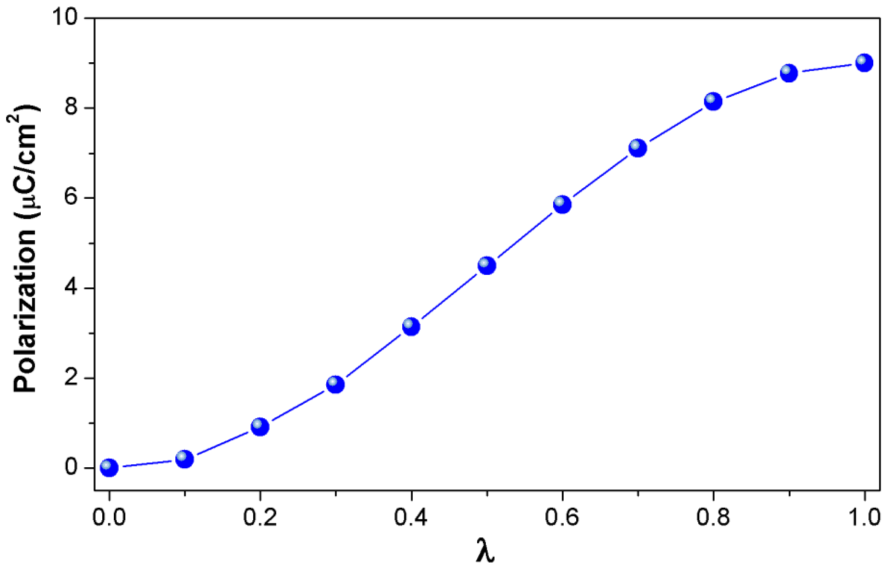

Supplementary Figure 5. Evolution of polarization during the phase transition of 1. The polarization as a function of λ was evaluated by density functional theory (DFT). The state of λ = 0 corresponds to the paraelectric phase, the state of λ = 1 corresponds to the ferroelectric phase, and the states of 0 < λ < 1 correspond to the transition states.

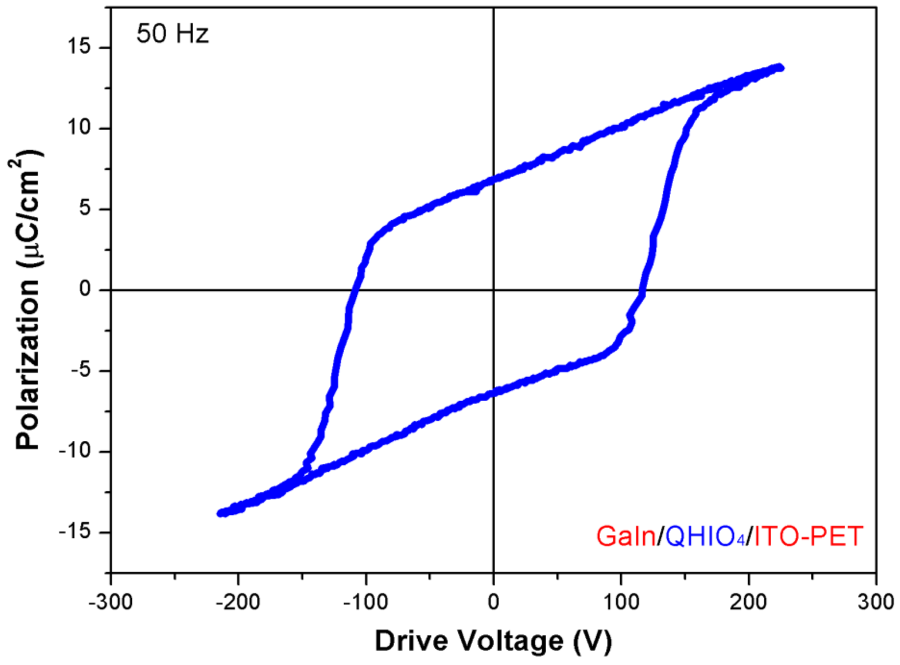

Supplementary Figure 6. Ferroelectric hysteresis loop. This was recorded on the thin film of **1** on an ITO coated flexible PET substrate.

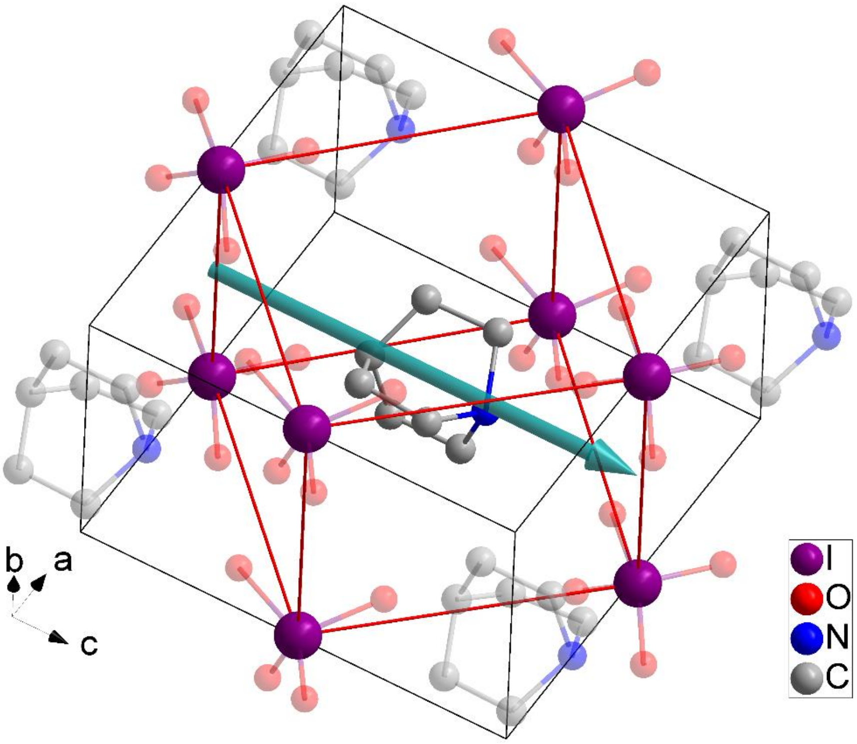

Supplementary Figure 7. The polarization direction of 1. The polarization direction (green arrow, [001]-direction) in the room temperature crystal cell is coincided with the [110]-direction of its cubic paraelectric phase unit cell (which is outlined in red solid line).

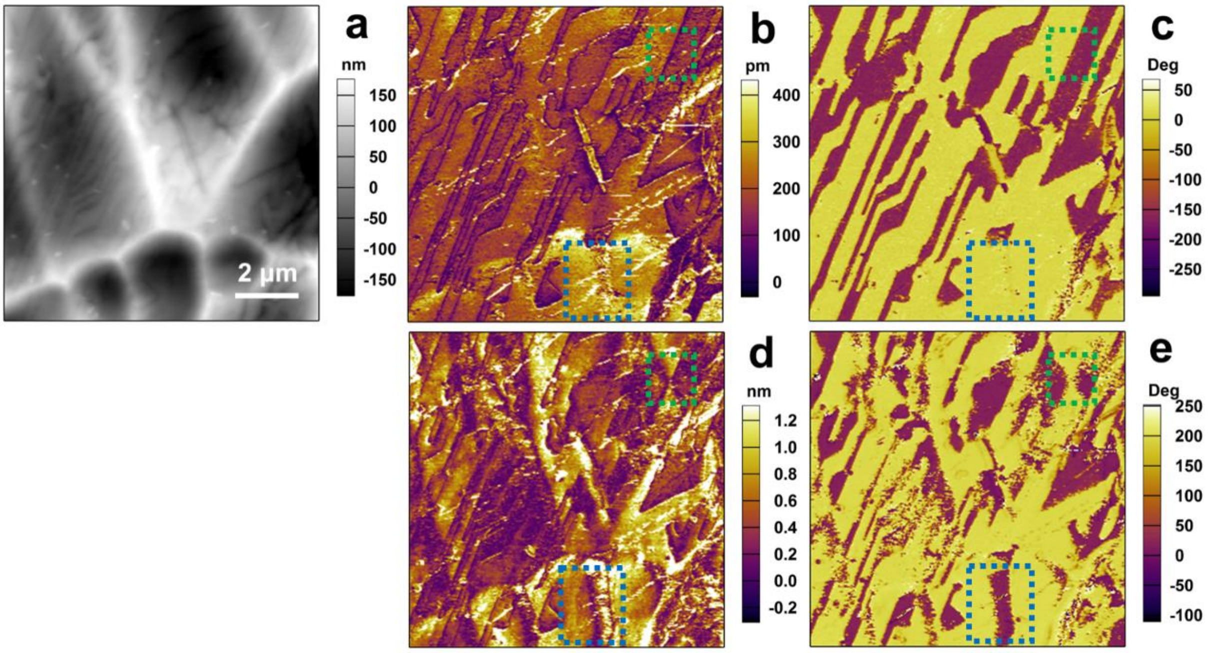

Supplementary Figure 8. PFM images of the film surface of 1. (**a**) The topographic image. (**b**,**c**) Vertical PFM images. (**d**,**e**) Lateral PFM images. It is notable that the domain distribution in the vertical mode is similar to, but not identical to that in the lateral mode. For instance, in the same region marked with green dashed rectangle, the vertical phase image presents stripe bipolar domain pattern, whereas the lateral one shows the shape of bipolar domain pattern like sand clock, implying the possible presence of 60° or 120° domains. Furthermore, there exists different distribution in the region marked with blue dashed rectangle. From the vertical phase image, we can see the single-domain state in this region, while distinct bipolar domain pattern emerges in the lateral phase image, suggesting the possible existence of 90° domains.

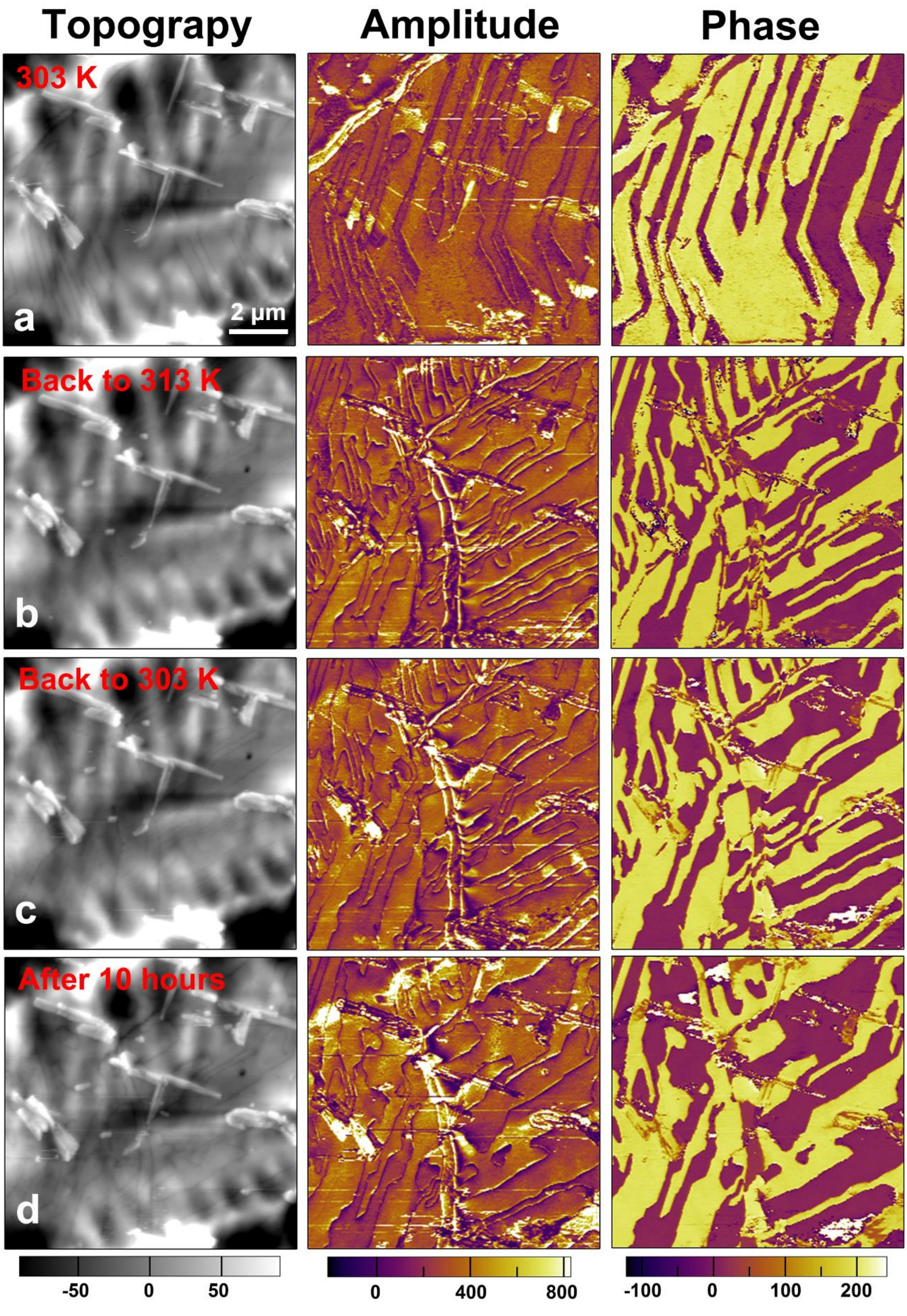

Supplementary Figure 9. Domain evolution of 1. This was recorded on the as-processed thin film of **1** in a heating and cooling process. The panels are arranged as the sequence： topography (left), VPFM amplitude (middle) and VPFM phase (right). (**a**) The initial state at 303 K. (**b**) The state heated to 333

K and then cooled back 313 K. (**c**) The state further cooled to 303 K. (**d**) The state after 10 hours. In (**a**), the thin film of **1** shows two types of representative domain structures at room temperature. As temperature increase to 333 K, the domain structure was vanishing, supporting the existence of a paraelectric phase above T_c. Then the sample was cooled down to below T_c, the domains with clear contrast are detectable again, which is attributed to the phase transition from paraelectric to ferroelectric phase. These series of PFM images present the evolution of the ferroelectric domain structures. The domain patterns in (**b**) and (**c**) are strikingly different from those in (**a**), because of the regrowth of ferroelectric domains induced by lowering temperature from the paraelectric phase. After aging for 10 hours, the domain structure shows little change.

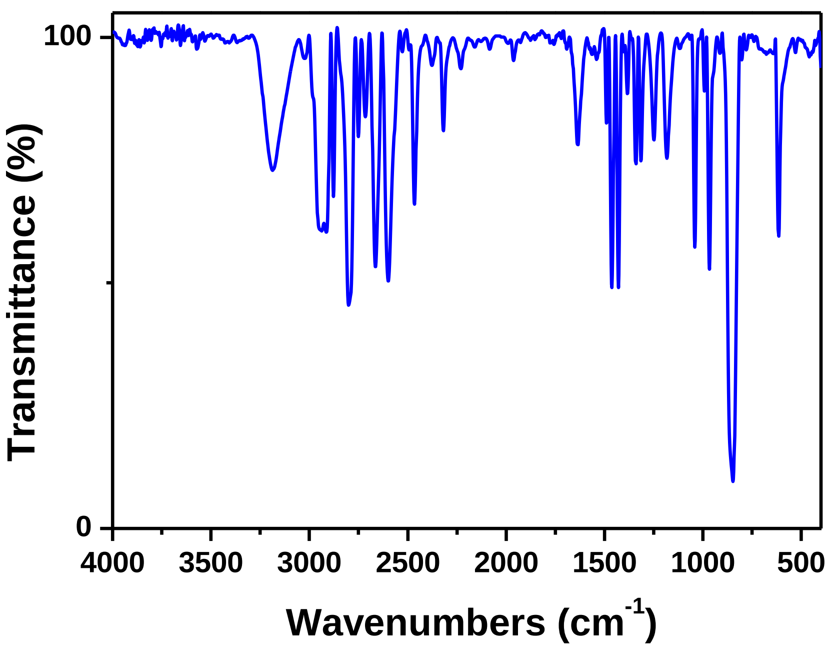

Supplementary Figure 10. IR spectrum for 1.

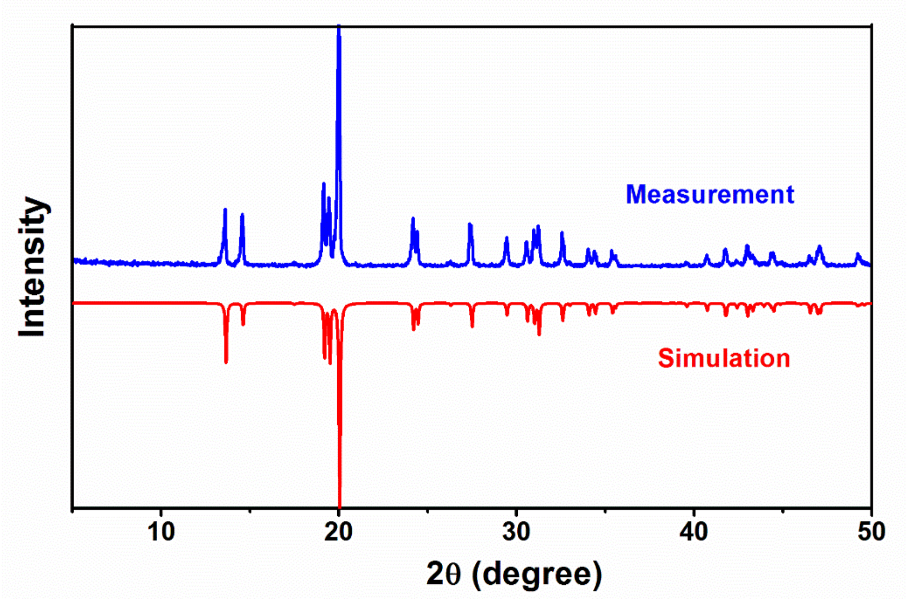

Supplementary Figure 11. Pattern of the powder X-ray diffraction (PXRD) of 1. It verifies the purity of the bulk phase.

Supplementary Table 1. Comparison of ferroelectric properties between 1 and quinuclidinium perrhenate.

	Remnant polarization ($\mu C/cm^2$)	Temperature range of ferroelectric phase (K)	Paraelectric phase	Ferroelectric phase	Number of polar-axes
**1**	6.2[1]<br>6.8[2]	<322	$Pm\bar{3}m$	$Pmn2_1$	6
Quinuclidinium perrhenate[3]	3.5	345-367	$Pm\bar{3}m$	$R3m$	4

1 Obtained on thin-film sample on ITO/glass substrate

2 Obtained on thin-film sample on flexible ITO/PET substrate

3 Harada, J. *et al.* Directionally tunable and mechanically deformable ferroelectric crystals from rotating polar globular ionic molecules. *Nat. Chem.* 946-952 (2016).

Supplementary Table 2. Crystal data for 1.

Compound	**1**	
Temperature	293 K	338 K
Formula,<br>*M*r	$C_7H_{14}NIO_4$<br>303.09	
Crystal system,<br>space group	orthorhombic<br>$Pmn2_1$	cubic,<br>*Pm*-3*m*
a, b, c (Å)	9.099(9)<br>6.059(6)<br>9.240(9)	6.437(8)<br>6.437(8)<br>6.437(8)
V (Å^3)	509.4(9)	266.7(10)
*D*c (g cm^{-3})	1.976	1.887
μ (mm^{-1})	3.128	2.0988
R_1 (I > 2σ(I))	0.0362	0.1450
wR_2 (all data)	0.0698	0.3087
S	1.144	1.402

Supplementary Table 3. Charge distribution in a unit cell of 1.

Atoms	Coordinate		Center coordinate
N	N1^{1}(0, 0.5460, 0.1438)	N1^{2}(1, 0.5460, 0.1438)	(0.5, 0.5, 0.6438)
	N1^{3}(0, 0.5460, 1.1438)	N1^{4}(1, 0.5460, 1.1438)	
	N2(0.5, 0.4540, 0.6438)		
I	I1^{1}(0, 1.0104, 0.5109)	I1^{2}(0, 0.0104, 0.5109)	(0.5, 0.5, 0.5109)
	I2^{1}(0.5, 0.9896, 0.0109)	I2^{2}(0.5, -0.0104, 0.0109)	
	I3^{1}(1, 1.01043, 0.5109)	I3^{2}(1, 0.0104, 0.5109)	
	I4^{1}(0.5, 0.9896, 1.0109)	I4^{2}(0.5, -0.0104,0.0109)	

Supplementary note 1

Point charge model analysis for polarization: According to the crystal structure data at 293 K, we select a unit cell and make an assumption that the positive charge of $(C_7H_{14}N)^+$ and the negative charge of $(IO_4)^-$ is on the N atom and I atom, respectively. The charge distribution is listed in Supplementary Table 3.

$P = \lim \frac{1}{V} \Sigma q_i r_i$

$= (q_N r_N + q_I r_I) / V$

$= [(e \times 0.6438) + (-e \times 0.5109)] \times 2 \times c/V$

$= [0.1329 \times 2 \times 1.6 \times 10^{-19} \times 9.24 \times 10^{-10}\ C\ m] / (509.4 \times 10^{-30}\ m^3)$

$= 7.71 \times 10^{-2}\ C\ m^{-2} = 7.71\ \mu C\ cm^{-2}$

Supplementary method

Berry phase calculation. In order to investigate the microscopic ferroelectric polarization, we carried out density functional calculations based on the Berry phase method developed by Kingsmith and Vanderbilt.[1,2] The first-principles calculations were performed within the framework of density functional theory (DFT) implemented in the Vienna ab initio Simulation Package (VASP).[3,4] The energy cut-off for the expansion of the wave functions was fixed to 550 eV and the exchange−correlation interactions were treated within the generalized gradient approximation of the Perdew−Burke−Ernzerhof type.[5] For the integrations over the k-space we used a 3×4×3 k-point mesh. The experimental room temperature crystal structure was used as the ground state for evaluating the ferroelectric polarization. The calculated polarization vector coincides with the crystallographic *c*-axis, which is in good agreement with the polar axis of the space group $Pmn2_1$. In order to further evaluate the magnitude of the ferroelectric polarization, we consider the path connecting the centric to the polar structure by continuously interpolating the atomic positions which involves the rotation of quinuclidinium cation relative to the periodate anion. The continuous evolution of spontaneous polarization from the centrosymmetric structure ($\lambda = 0$) to the polar structure ($\lambda = 1$) is plotted as a function of dimensionless parameter λ in Figure S1. The dimensionless parameter λ is the normalized amplitude of the atomic distortion connecting the structure of the paraelectric phase and the structure of the ferroelectric phase. From the curve, the estimated value is ~ 9.01 $\mu C/cm^2$ in the ferroelectric structure, which are mainly stemmed from the misalignment of the positive and negative centers. The additional considerations of molecular dipoles and electron polarization by Berry phase method may be the main reason for explaining the value of the spontaneous polarization is slightly bigger than that of point charge model calculation.

Supplementary References

1 Kingsmith, R. D. & Vanderbilt, D. Theory of polarization of crystalline solids. *Phys. Rev. B* **47**, 1651-1654 (1993).

2 Vanderbilt, D. & Kingsmith, R. D. Electric polarization as a bulk quantity and its relation to surface-charge. *Phys. Rev. B* **48**, 4442-4455 (1993).

3 Kresse, G. & Furthmuller, J. Efficient iterative schemes for ab-initio total-energy calculations using a plane-wave basis set. *Phys. Rev. B* **54**, 11169-11186 (1996).

4 Kresse, G. & Furthmuller, J. Efficiency of ab-initio total energy calculations for metals and semiconductors using a plane-wave basis set. *Comput. Mater. Sci.* **6**, 15-50 (1996).

5 Perdew, J. P., Burke, K. & Ernzerhof, M. Generalized gradient approximation made simple. *Phys. Rev. Lett.* **77**, 3865-3868 (1996).
